# Duration of antibiotic therapy for bacteremia: a systematic review and meta-analysis

**DOI:** 10.1186/cc10545

**Published:** 2011-11-15

**Authors:** Thomas C Havey, Robert A Fowler, Nick Daneman

**Affiliations:** 1 Department of Medicine, University of Toronto, 1 Kings College Circle, O, M5S 1A8, Canada; 2 Department of Critical Care Medicine, Sunnybrook Health Sciences Centre, 2075 Bayview Avenue, University of Toronto, M4N 3N5 Canada; 3 Division of Infectious Diseases, Sunnybrook Health Sciences Centre, 2075 Bayview Avenue, University of Toronto, M4N 3N5 Canada

## Abstract

**Introduction:**

The optimal duration of antibiotic therapy for bloodstream infections is unknown. Shorter durations of therapy have been demonstrated to be as effective as longer durations for many common infections; similar findings in bacteremia could enable hospitals to reduce antibiotic utilization, adverse events, resistance and costs.

**Methods:**

A search of the MEDLINE, EMBASE and COCHRANE databases was conducted for the years 1947-2010. Controlled trials were identified that randomized patients to shorter versus longer durations of treatment for bacteremia, or the infectious foci most commonly causing bacteremia in critically ill patients (catheter-related bloodstream infections (CRBSI), intra-abdominal infections, pneumonia, pyelonephritis and skin and soft-tissue infections (SSTI)).

**Results:**

Twenty-four eligible trials were identified, including one trial focusing exclusively on bacteremia, zero in catheter related bloodstream infection, three in intra-abdominal infection, six in pyelonephritis, thirteen in pneumonia and one in skin and soft tissue infection. Thirteen studies reported on 227 patients with bacteremia allocated to 'shorter' or 'longer' durations of treatment. Outcome data were available for 155 bacteremic patients: neonatal bacteremia (*n *= 66); intra-abdominal infection (40); pyelonephritis (9); and pneumonia (40). Among bacteremic patients receiving shorter (5-7 days) versus longer (7-21 days) antibiotic therapy, no significant difference was detected with respect to rates of clinical cure (45/52 versus 47/49, risk ratio 0.88, 95% confidence interval [CI] 0.77-1.01), microbiologic cure (28/28 versus 30/32, risk ratio 1.05, 95% CI 0.91-1.21), and survival (15/17 versus 26/29, risk ratio 0.97, 95% CI 0.76-1.23).

**Conclusions:**

No significant differences in clinical cure, microbiologic cure and survival were detected among bacteremic patients receiving shorter versus longer duration antibiotic therapy. An adequately powered randomized trial of bacteremic patients is needed to confirm these findings.

## Introduction

A recent global point prevalence survey of infections in 1,265 intensive care units (ICUs) documented bloodstream infection (BSI) among 15% of patients [1], and this rate may be increasing over time because of increased use of immunosuppressive drugs, invasive procedures, and older patients who have concomitant medical conditions and who are admitted to intensive care [2,3]. These infections are a major contributor to patient morbidity [1] and are associated with a doubling or even tripling of mortality [4,5]. Mortality rates may be higher if delayed [6] or ineffective [7] initial antimicrobial therapy is prescribed, and so it is difficult to reduce broad-spectrum antibiotic use in the initial empiric phase of treatment in this vulnerable patient population [8]. In contrast, it may be more feasible to reduce antibiotic use at the back end of treatment courses. Up to half of the antibiotic use in hospital wards and critical care units is unnecessary or inappropriate, and excessive durations of treatment are the greatest contributor to inappropriate use [9-11]. A reduction in the length of antibiotic courses is, therefore, a potentially viable strategy to minimize the consequences of antibiotic overuse in critical care, including antibiotic resistance, adverse effects, *Clostridium difficile *colitis, and costs [12].

The optimal duration of therapy for primary BSI and BSI secondary to major organ system infections has been poorly defined. A review of the Infectious Diseases Society of America (IDSA) guidelines for the treatment of infections most commonly encountered in the critical care setting - including guidelines for community- and hospital-acquired pneumonia [13,14], intra-abdominal infection [15], catheter-related bloodstream infection (CRBSI) [16], pyelonephritis [17], and skin and soft tissue infection (SSTI) [18] - provides no guidance about the optimal duration of therapy for the subset of patients with bacteremia. In the absence of high-grade evidence, there is wide variability in antibiotic treatment duration recommendations from infectious disease and critical care specialists, and the presence of bacteremia is often used as a justification for extended courses of antibiotic therapy regardless of the observed clinical response to treatment [19,20]. Randomized controlled trials (RCTs) examining duration of therapy in several organ system infections have demonstrated that treatment can be shortened to 1 week or less without worsening patient outcomes [11,21-23], so it is plausible that treatment duration could potentially be shortened for BSIs as well.

The objective of this study was to determine whether the therapeutic equivalence of shorter- and longer-course antibiotic therapy extends to patients with bacteremia. We conducted a systematic review and meta-analysis of RCTs explicitly examining the efficacy of shorter-course versus longer-course antibiotic therapy for patients with bacteremia as well as comparable trials examining the organ system infections most commonly causing bacteremia in critically ill patients.

## Materials and methods

### Search strategy

We searched the Cochrane Central Register of Controlled Trials, Ovid Medline (1948 to 2010), and Ovid Embase (1947 to 2010) to find relevant RCTs comparing shorter versus longer durations of treatment for bacteremia or the five most common organ system infections causing bacteremia in critically ill patients [1]. Filters for RCTs specified by the Scottish Intercollegiate Guidelines Network [24] were combined individually with the following keywords: 'bacteremia', 'bacteraemia', 'pneumonia', 'pyelonephritis', 'cellulitis', 'soft tissue infection', 'skin disease, bacterial', 'peritonitis', 'intra-abdominal infection', 'catheter-related infections', and 'catheterization, central venous AND bacteremia OR bacteraemia OR bloodstream infection'. Results were restricted to trials performed on humans. We manually reviewed the reference lists of retrieved studies, editorials, systematic reviews, and meta-analyses to identify additional relevant trials.

### Eligibility criteria

Clinical trials were included if they used random allocation of patients to treatment groups comparing differing durations of oral, intramuscular, or intravenous antimicrobial treatment of bacteremia, CRBSIs, pneumonia, pyelonephritis, SSTI, or intra-abdominal infections. Eligible trials randomly assigned subjects to one of two different durations of treatment with the same antibiotic regimen and evaluated one or more of the following outcomes: clinical cure, microbiologic cure, or survival. We excluded trials that determined duration of treatment on the basis of physician discretion, clinical improvement, or biomarker measurements. Observational studies were not included, because a large volume of studies was anticipated and because the association of treatment duration and patient outcomes would be impossible to interpret in light of survivor bias and bias-by-indication. A sample of 200 citations from each of the six searches (1,200 total citations) was independently reviewed by a second author to assess agreement via calculation of a kappa statistic. Disagreements were resolved through consensus.

### Study quality

The quality of included trials was appraised by using the Cochrane Risk of Bias tool, which assesses sequence generation, allocation concealment, blinding, data completeness, and outcome reporting, and, on the basis of these domains, summarizes studies as exhibiting a low, high, or unclear risk of bias.

### Data collection

Data were collected via a prespecified data extraction spreadsheet with criteria agreed upon by all authors. The information extracted from each trial consisted of the following: (1) infectious syndrome; (2) number of eligible patients screened and randomly assigned; (3) patient characteristics; (4) antibiotic regimen; (5) shorter- and longer-arm treatment duration; (6) day of randomization; (7) allocation sequence method, method of concealment, and presence or absence of blinding strategy; (8) inclusion and exclusion criteria; (9) extent of loss to follow-up; (10) primary outcome measure (including clinical cure, microbiologic cure, and survival); and (11) results of primary outcome in shorter- and longer-arm treatment groups and bacteremic subgroups (CRBSI, pneumonia, intra-abdominal infection, pyelonephritis, and SSTI).

### Outcomes

From all trials, we extracted available data on outcomes of clinical cure, microbiologic cure, and survival for the overall groups of patients receiving shorter- and longer-duration antibiotic therapy. In our primary analysis, though, we examined these three outcomes (clinical cure, microbiologic cure, and survival) among the subgroup of patients with bacteremia in each treatment arm. Therefore, individual studies were closely examined for any information relating to the collection of blood cultures and stated inclusion/exclusion of patients with bacteremia. In studies that included patients with bacteremia, data regarding the proportion with bacteremia in each intervention arm (if available) and outcomes for this bacteremic subgroup (if available) were extracted.

### Statistical analysis

Outcomes with shorter versus longer antibiotic therapy were reported as risk differences and relative risks with 95% confidence intervals (CIs). In primary analyses, these measures of effect were calculated for the bacteremic subgroups. In secondary analyses, these measures of effect were calculated for the overall study populations. Heterogeneity was assessed across all studies (and for studies within each individual syndrome) via graphical inspection of forest plots as well as calculation of I^2 ^and chi-squared statistics. *P *values of less than 0.1 were deemed to suggest statistically significant between-study heterogeneity [25]. Pooled relative risks were calculated by using the Mantel-Haenszel fixed effects model. These statistical analyses were performed by using Review Manager version 5 software (The Cochrane Collaboration, Copenhagen, Denmark).

## Results

### Study selection and characteristics

**Figure 1:**
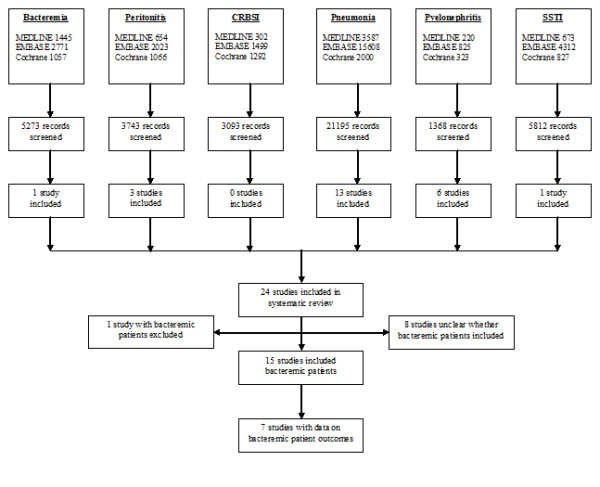
**Flowchart describing citations screened, studies included for searches of bacteremia, and infectious syndromes most commonly causing bacteremia**. CRBSI, catheter-related bloodstream infection; SSTI, skin and soft tissue infection.

Our search strategy identified 40,484 total references in six separate searches. A total of 24 trials met inclusion criteria, and there was excellent agreement between investigators (kappa statistic = 0.90). Only 11 out of 24 studies reported on allocation concealment, 13 out of 24 employed some form of blinding, 10 out of 24 used placebo, and 9 out of 24 had a low risk of bias on the basis of clear reporting of all three of these factors (Table 1). These trials consisted of 1 trial dedicated to bacteremia and 23 trials examining the syndromes most commonly causing critical care-associated bacteremia (0 in CRBSI, 3 in intra-abdominal infection, 6 in pyelonephritis, 1 in SSTI, and 13 in pneumonia) (Figure 1). Fifteen studies (63%) included patients with bacteremia, and 13 studies (54%) reported on the proportion of patients with bacteremia (1 out of 1 trial in bacteremia, 0 out of 0 in CRBSI, 2 out of 3 in intra-abdominal infection, 2 out of 6 in pyelonephritis, 0 out of 1 in SSTI, and 8 out of 13 in pneumonia).

**Table 1:** Randomized trials of shorter- versus longer-duration antibiotic therapy in bacteremia or syndromes commonly causing bacteremia.

Author	Syndrome	Number screened (number randomly assigned)	Population	Antibiotic	Short arm, days	Long arm, days	Day of random assignment	Random assignment/Concealment	Blinding/Placebo	Patients with bacteremia
Chowdhary *et al. *[26]	Bacteremia	229 (69)	Hospitalized neonates	Culture-directed	7	14	7	Computer-generated/Yes	Yes/No	Included

Basoli *et al. *[27]	Peritonitis	111 (90)	Secondary peritonitis	Ertapenem	3	5-14	3	Unclear/Unclear	Yes/Yes	Unclear

Runyon *et al. *[28]	Peritonitis	140 (100)	Hospitalized adults; SBP	Cefotaxime	5	10	1	Unclear/No	No/No	Included

Chaudhry *et al. *[29]	Peritonitis	60 (50)	Hospitalized adults; SBP	Cefoperazone	5	10	1	Unclear/No	No/No	Included

Hepburn *et al. *[49]	SSTI	169 (87)	Outpatient adults	Levofloxacin	5	10	5	Computer-generated/Yes	Yes/Yes	Excluded

De Gier *et al. *[43]	Pyelonephritis	N/A (54)	Hospitalized adults; complicated UTI	Fleroxacin	7	14	1	Unclear/Unclear	Unclear/No	Unclear

Stamm *et al. *[44]	Pyelonephritis	98 (60)	Outpatient adult women	Ampicillin or TMP/SMX	14	42	1	Random number table/No	No/No	Unclear

Gleckman *et al. *[45]	Pyelonephritis	N/A (54)	Hospitalized adult women	Gentamicin/tobramycin; ampicillin, TMP/SMX, cephalexin	10	21	1	Random number table/No	No/No	Unclear

Pylkkanen *et al. *[46]	Pyelonephritis	271 (149)	Infants and children	Sulfafurazole	10	42	1	Unclear/No	No/No	Unclear

Jernelius *et al. *[47]	Pyelonephritis	N/A (77)	Outpatient and hospitalized adults	Pivampicillin and pivmecillinam	7	21	1	Computer-generated/Yes	Yes/Yes	Included

Cheng *et al. *[48]	Pyelonephritis	N/A (80)	Hospitalized children	Acc. to culture	14	21	1	Serial entry/Unclear	Unclear/No	Included

Engle *et al. *[30]	Pneumonia	51 (26)	Hospitalized neonates	Ampicillin and gentamicin	2	4	2	Unclear/Unclear	Unclear/No	Included

Engle *et al. *[31]	Pneumonia	212 (73)	Hospitalized neonates	Ampicillin and gentamicin	4	7	2	Unclear/Yes	Unclear/No	Included

ISCAP [33]	Pneumonia	N/A (2,188)	Outpatient children 2 to 59 months	Amoxicillin	3	5	1	Unclear/Yes	Yes/Yes	Unclear

MASCOT [34]	Pneumonia	N/A (2,000)	Outpatient children 2 to 59 months	Amoxicillin	3	5	1	Computer-generated/Yes	Yes/Yes	Unclear

Vuori-Holopainen *et al. *[32]	Pneumonia	178 (72)	Hospitalized children 3 months to 15 years	Penicillin or cefuroxime	4	7	1	Computer-generated/No	No/No	Included

Tellier *et al. *[36]	Pneumonia	581 (575)	Outpatient and hospitalized adults	Telithromycin	5	7	1	Unclear/Yes	Yes/Yes	Included

File *et al. *[35]	Pneumonia	N/A (512)	Outpatient adults	Gemifloxacin	5	7	1	Unclear/Unclear	Yes/Unclear	Included

Dunbar *et al. *[37]	Pneumonia	2,521 (530)	Outpatient and hospitalized adults	Levofloxacin	5	10	1	Unclear/Yes	Yes/Yes	Included

Leophonte *et al. *[39]	Pneumonia	N/A (244)	Hospitalized adults	Ceftriaxone	5	10	1	Unclear/Yes	Yes/Yes	Included

Siegel *et al. *[38]	Pneumonia	N/A (52)	Hospitalized adults	Cefuroxime	7	10	1	Computer-generated/Yes	Yes/Yes	Included

El Moussaoui *et al. *[40]	Pneumonia	186 (121)	Hospitalized adults	Amoxicillin	3	8	3	Unclear/Yes	Yes/Yes	Included

Chastre *et al. *[42]	Pneumonia	1,171 (402)	Hospitalized adults	Culture-directed	8	15	3	Computer-generated/Yes	Yes/No	Included

Fekih Hassen *et al. *[41]	Pneumonia	39 (30)	Hospitalized adults	Culture-directed	7	10	2	Random number table/No	No/No	Unclear

ISCAP, IndiaClen Short Course Amoxicillin Pneumonia; MASCOT, Multicentre Amoxicillin Short Course Therapy; N/A, not applicable; SBP, spontaneous bacterial peritonitis; SSTI, skin and soft tissue infection; TMP/SMX, trimethoprim/sulfamethoxazole; UTI, urinary tract infection.

### Outcomes among the overall study populations

The included studies involved 7,695 participants of varying ages, syndromes, and definitions of shorter and longer therapy (Table 1). Clinical cure (Additional file 1), microbiologic cure (Additional file 2), and survival (Additional file 3) were similar for overall study populations receiving shorter- versus longer-duration therapy. Significant heterogeneity across the different infectious syndromes was detected for outcomes of microbiologic cure (I^2 ^= 71%, *P *value < 0.0001) but not for clinical cure (I^2 ^= 0%, *P *= 0.68) or survival (I^2 ^= 0%, *P *= 0.64). Within individual syndromes, there was evidence of between-study heterogeneity for pyelonephritis but not for studies of the other bacteremic syndromes.

### Outcomes among patients with bacteremia

**Figure 2:**
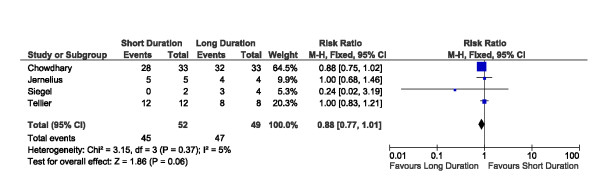
**Forest plot for outcome of clinical cure among bacteremic subgroups of randomized trials of shorter versus longer antibiotic treatment**. CI, confidence interval; df, degrees of freedom; M-H, Mantel-Haenszel.

**Figure 3:**
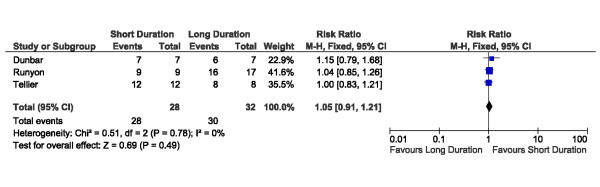
**Forest plot for outcome of microbiologic cure among bacteremic subgroups of randomized trials of shorter versus longer antibiotic treatment**. CI, confidence interval; df, degrees of freedom; M-H, Mantel-Haenszel.

**Figure 4:**
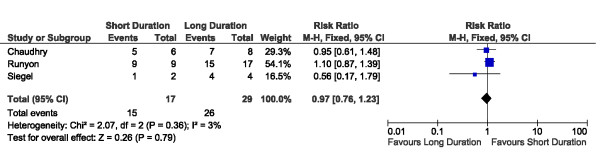
**Forest plot for outcome of survival among bacteremic subgroups of randomized trials of shorter versus longer antibiotic treatment**. CI, confidence interval; df, degrees of freedom; M-H, Mantel-Haenszel.

A total of 227 patients with documented bacteremia were described across the included studies (Table 2). Treatment outcomes for patients with bacteremia were available from 7 of these 11 trials (64%), contributing outcome data for a total of 155 patients with documented positive blood cultures (Table 2). In our primary study analyses, we compared outcomes among bacteremic patients receiving shorter-duration (ranging from 5 to 7 days) versus longer-duration (ranging from 7 to 21 days) antibiotic therapy. Among bacteremic patients receiving shorter versus longer antibiotic therapy, no significant difference was detected with respect to rates of clinical cure (45/52 versus 47/49, risk ratio 0.88, 95% CI 0.77 to 1.01) (Figure 2), microbiologic cure (28/28 versus 30/32, risk ratio 1.05, 95% CI 0.91 to 1.21) (Figure 3), or survival (15/17 versus 26/29, risk ratio 0.97, 95% CI 0.76 to 1.23) (Figure 4). No significant heterogeneity between studies was detected for clinical cure (I^2 ^= 5%, *P *= 0.37), microbiologic cure (I^2 ^= 0%, *P *= 0.78), or survival (I^2 ^= 3%, *P *= 0.36).

**Table 2:** Randomized controlled trials of shorter versus longer antibiotic therapy that included patients with bacteremia.

Author	Syndrome	All patients blood cultured?	Short-arm group, number (percentage) bacteremic	Long-arm group, number (percentage) bacteremic
Chowdhary *et al. *[26]	Non-*Staphylococcus aureus *bacteremia	Yes	26/26 (100%)	26/26 (100%)
	*S. aureus *bacteremia	Yes	7/7 (100%)	7/7 (100%)
Runyon *et al. *[28]	Peritonitis	Yes	9/43 (20.9%)	17/47 (36.2%)
Chaudhry *et al. *[29]	Peritonitis	Yes	6/25 (24%)	8/25 (32%)
Jernelius *et al. *[47]	Pyelonephritis	Yes	5/32 (15.6%)	4/29 (13.8%)
Cheng *et al. *[48]	Pyelonephritis	Yes	1/41 (2.4%)	3/39 (7.7%)
Engle *et al. *[30]	Pneumonia	Yes	0/14 (0%)	0/12 (0%)
Engle *et al. *[31]	Pneumonia	Yes	0/35 (0%)	0/38 (0%)
Vuori-Holopainen *et al. *[32]	Pneumonia	Yes	N/A	N/A
Tellier *et al. *[36]	Pneumonia	Yes	14/187 (7.5%)	9/191 (4.7%)
File *et al. *[35]	Pneumonia	Yes	N/A	N/A
Dunbar *et al. *[37]	Pneumonia	Yes	7/256 (2.7%)	7/272 (2.6%)
Leophonte *et al. *[39]	Pneumonia	Yes	11/125 (8.8%)	12/119 (10.1%)
Siegel *et al. *[38]	Pneumonia	Yes	2/24 (8.3%)	4/22 (18.2%)
El Moussaoui *et al. *[40]	Pneumonia	Unclear	6/56 (10.7%)	8/63 (12.7%)
Chastre *et al. *[42]	Pneumonia	Yes	14/197 (7.1%)	14/204 (6.9%)

N/A, not applicable.

### Detailed outcomes among patients with bacteremia according to underlying infectious syndromes

#### Trials focusing exclusively on bacteremia

A single randomized trial examined duration of antibiotic therapy exclusively in bacteremia. Chowdhary and colleagues [26] randomly assigned ICU neonates with positive blood cultures and no obvious deep-seated focus of infection to 7 or 14 days of culture-directed antibiotic therapy. Patients were randomly assigned at day 7 if symptoms had clinically remitted by day 5 (32.5% were excluded because of failure to remit). The composite outcome included bacteriologic relapse with the same organism, a recurrent episode of illness with an elevation in serum C-reactive protein, or a subsequent clinical diagnosis of sepsis by a blinded adjudication committee. No statistically significant difference in treatment outcomes between groups was reported, and 28 out of 33 patients (84.8%) in the 7-day arm and 32 out of 33 (97%) in the 14-day arm were successfully treated. In a preplanned subgroup analysis, the success rate for *Staphylococcus aureus *bacteremia was lower with shorter versus longer therapy: 3 out of 7 (42.9%) versus 7 out of 7 (100%), *P *= 0.02. Treatment success was not significantly different for shorter and longer duration of therapy in non-*S. aureus *infections (25 out of 26 in both groups).

#### Catheter-related bloodstream infection

A search of 3,093 indexed citations from Medline, Embase, and the Cochrane Database did not yield any eligible randomized trials examining duration of antibiotic therapy in CRBSI.

#### Intra-abdominal infection

Three randomized trials examined duration of antibiotic therapy in intra-abdominal infection. The first randomly assigned 90 adult patients with community-acquired localized secondary intra-abdominal infections of mild to moderate severity to 3 or at least 5 days (mean of 5.7 days and range of 5 to 10 days) of postoperative ertapenem [27]. Patients were included if symptoms and leukocytosis had improved by day 3. Clinical cure was achieved in 39 out of 42 patients (92.9%) receiving shorter therapy and in 43 out of 48 (89.6%) receiving longer therapy. Although blood cultures were drawn when 'clinically indicated', the prevalence of bacteremia and outcomes in patients with bacteremia were not reported [27].

Two unblinded randomized trials [28,29] compared 5 versus 10 days of cephalosporin therapy in patients with spontaneous bacterial peritonitis (SBP). All patients underwent blood cultures at enrollment. In one trial, 26 out of 90 (28.9%) were bacteremic, and no significant difference in survival was observed for shorter (43 out of 43) versus longer (45 out of 47) treatment [28]. Infection-related mortality among bacteremic patients treated for 5 days (0%) and 10 days (11.8%) was not significantly different [28]. In the second trial, 14 out of 50 (28%) were bacteremic, and no significant difference in outcome was observed; 83.3% of short-arm and 87.5% of long-arm patients survived their infections [29]. Secondary outcomes of relapse and hospitalization mortality were also similar between treatment arms and for bacteremic subgroups [28,29].

#### Pneumonia

Thirteen eligible trials that included a total of 6,825 patients of various ages, clinical settings, and treatments were included (Table 1). Five trials examined treatment duration in pediatric pneumonia, six studied adult community-acquired pneumonia (CAP), and two studied ventilator-associated pneumonia (VAP).

Clinical success rates were similar with shorter versus longer therapy in five studies of neonatal pneumonia (Table 1 and Figure 2) [30-34]. These studies either documented no patients with bacteremia [30,31] or did not report on this subgroup [32-34].

Six trials of adult CAP found no difference in clinical effectiveness of shorter versus longer duration of treatment [35-40]. Five trials included information on patients with bacteremia [36-40], and three provided subgroup analyses of outcomes of patients with bacteremia [36-38]. In a trial of mild to moderate CAP, 388 patients were randomly assigned to 5 or 7 days of treatment with telithromycin [36]. Blood cultures were positive in 23 out of 388 (5.2%), and treatment was successful for 20 out of 20 patients with *Streptococcus pneumoniae *bacteremia. The pathogens implicated in the remaining three bacteremias (and the corresponding patient outcomes) were not reported. In a trial of 530 patients with mild to moderate CAP [37], only 14 (2.7%) were bacteremic, and no significant difference in clinical success was noted between 7 out of 7 bacteremic patients receiving 5 days of levofloxacin and 6 out of 7 (85.7%) receiving 10 days. Finally, a trial (*n *= 52) of adult inpatients with CAP documented bacteremia in 6 out of 46 patients (13%). Neither of 2 bacteremic patients receiving 7 days of cefuroxime achieved clinical success (although 1 died within 24 hours of initiation) versus 3 out of 4 patients receiving 10 days [38].

One small VAP trial randomly assigned 30 patients to 7 or 10 days of culture-directed antibiotic therapy, and similar rates of survival among those treated for 7 days (64.3%) and 10 days (62.5%) were reported [41]. The numbers of patients with bacteremia or their outcomes were not provided [41]. In a large, multicenter, high-quality trial that randomly assigned 402 adult patients to 8 or 15 days of therapy [42], survival was not different: 160 out of 197 (81.2%) versus 169 out of 204 (82.8%) [42]. While all patients were blood cultured at enrollment and 28 out of 402 patients (7%) were bacteremic, the outcomes of these patients with bacteremia were not separately reported [42].

#### Pyelonephritis

Six trials of treatment duration for pyelonephritis were included [43-48]. Only two out of six trials reported outcomes for patients with bacteremia [47,48]. In a blinded, placebo-controlled trial, 77 adults with pyelonephritis were randomly assigned to 7 versus 21 days of treatment with pivampicillin and pivmecillinam [47]. All patients underwent blood cultures at enrollment, and 9 out of 61 (14.8%) were bacteremic [47]. Clinical cure was achieved in 4 out of 4 bacteremic patients receiving 7 days and 5 out of 5 bacteremic patients receiving 21 days of treatment [47]. A second trial involved 14 versus 21 days of antibiotics for 80 children with acute lobar nephronia, defined as an advanced state of pyelonephritis which may progress to renal abscess if left untreated [48]. Clinical cure was lower among patients receiving shorter versus longer therapy (34 out of 41 versus 39 out of 39, *P *= 0.01) [48]. Four patients were bacteremic, but their outcomes were not provided [48].

#### Skin and soft tissue infection

A single randomized trial of antibiotic duration in SSTI was identified [49]. Adult patients with uncomplicated SSTIs were randomly assigned to 5 or 10 days of therapy with levofloxacin but only if they were improving after 5 days of treatment [49]. Clinical cure was observed in 43 out of 44 patients (97.7%) receiving 5 days of therapy and in 42 out of 43 (97.7%) receiving 10 days [49]. Blood cultures were drawn from eight patients when bacteremia was suspected and were positive in one patient, who was excluded from the trial [49].

## Discussion

This systematic review and meta-analysis identified only one RCT examining shorter versus longer duration of antibiotic therapy exclusively for patients with bacteremia and a further 23 trials among patients with the infectious syndromes that are most commonly associated with bacteremia in the ICU. No significant difference in cure or survival was detected for bacteremic patients receiving shorter- versus longer-duration therapy. These data indicate that shorter-duration therapy may be effective for BSIs but also highlight the need for a larger, high-quality trial dedicated to this question.

The only trial randomly assigning exclusively patients with bacteremia, rather than an identified infectious syndrome, was conducted in a severely ill neonatal population [26]. The main finding of this trial was that overall outcomes were not significantly different for neonates receiving shorter- versus longer-duration antibiotics. A high rate of failure was seen among the small number of patients receiving short-duration treatment for *S. aureus *bacteremia, and this is consistent with the findings of some prior retrospective studies and current treatment guidelines for this pathogen [50,51]; yet other studies support short-course therapy for catheter-associated *S. aureus *bacteremia [52]. This review highlights the potential importance of considering *S. aureus *bacteremia separately from other pathogens in the context of adequately powered trials in the future.

Although bacteremia caused by intravascular catheters is often cited as the commonest cause of BSI in the critical care setting, we were unable to identify any prospective, randomized investigations examining the duration of antibiotic therapy for CRBSI. Recommendations for length of treatment from the current IDSA guidelines are based only on expert opinion and retrospective case series and call for 5 to 7 days for infections caused by coagulase-negative staphylococci, 7 to 14 days for *Enterococci *and Gram-negative organisms, and 2 to 6 weeks for *S. aureus *[16]. Trials are urgently needed, as this may be the syndrome most appropriate for shorter-course therapy given that the focus of infection is removable and thereby leaves no persisting infectious nidus for most patients.

The available trials randomly assigning patients with intra-abdominal infections to shorter versus longer durations of antibiotic therapy were conducted in non-ICU settings and populations and explicitly excluded those with generalized secondary peritonitis or nosocomial infections. Equivalent outcomes for shorter- versus longer-duration therapy for SBP and localized intra-abdominal infection are in keeping with similar findings in a retrospective analysis of 929 patients with intra-abdominal infections, in which less than 7 days of therapy was not associated with higher complications or mortality [53]. We have not uncovered evidence, though, of whether the effectiveness of short-duration therapy extends to patients with severe infections complicated by bacteremia or in those for whom source control cannot readily be achieved.

Several narrative reviews [54,55] and meta-analyses [22,23] of RCTs have provided evidence of the efficacy and safety of treating mild to moderate CAP with short-duration antibiotic therapy (5 to 7 days). The 13 studies of pneumonia identified for this review included 5 studies of CAP that provided the prevalence of bacteremia; in total, 80 patients with bacteremia were randomly assigned in equal numbers to short (3 to 7 days) and long (7 to 10 days) durations of therapy. Only three studies provided outcome data for bacteremic subgroups, and clinical cure was reported in 19 out of 21 patients (89.4%) receiving short-duration therapy (5 to 7 days) and in 17 out of 19 patients (90.4%) receiving extended therapy (7 to 10 days). These results provide a modicum of support for the growing consensus that CAP may be safely treated with shorter durations of therapy irrespective of the presence of bacteremia [11,12]. Although a large VAP trial documented equivalent survival with shorter (8 days) versus longer (15 days) treatment [42], very few of these patients were bacteremic, and it is unclear whether shorter-duration treatment can be extended to this subgroup.

The eligible trials of treatment duration in pyelonephritis involved diverse patient populations, durations of treatment, and outcome measures, and this explains the heterogeneity of outcomes with shorter durations of therapy. Recently, the results of several RCTs have demonstrated short-course (5 or 7 days) fluoroquinolone therapy to be equally as efficacious as 10 to 14 days of treatment with comparator medications [56-58]. IDSA guidelines were accordingly amended to recommend therapy for 7 days [17], but the issue of bacteremia was not explicitly addressed. Our data suggest that patients with bacteremia secondary to uncomplicated pyelonephritis can be successfully treated with shorter-duration therapy.

The sole trial of treatment duration in SSTI demonstrated that, in a carefully selected healthy adult population, a short duration of therapy was associated with a cure rate equal to that of a long duration of therapy [49]. However, the explicit exclusion of patients with more serious infections, argues that it is unlikely that these data can be directly extrapolated to critically ill patients with bacteremic soft tissue infections.

The present review has several important limitations. The prespecified search strategy excluded unpublished data and non-English language trials. Of eligible trials, bias may have been introduced by low rates of blinding and use of placebo controls. Some studies included only patients with early clinical improvement for randomization to short- or long-course therapy; consequently, the effect size and findings may not be generalizable to sicker patient populations. Other trials excluded patients post-randomization or presented only per-protocol analyses and so may have excluded bacteremic patients failing therapy. Treatment outcomes for patients with bacteremia in individual studies were derived from small *post hoc *subgroup analyses. Finally, within individual infectious syndromes, significant variability was encountered both in study design and in the durations of therapy employed (with 14 days even considered shorter-course therapy in one study). However, the lack of outcome heterogeneity between syndromes suggests that it is valid to pool BSI data from multiple infectious foci into a single meta-analysis or enroll such patients within a single RCT.

## Conclusions

The ICU is the epicenter of bacteremia, antibiotic use, and antibiotic resistance in most hospitals. Reductions in the length of antibiotic treatment courses could potentially limit antibiotic use, adverse effects, and resistance pressure, but antibiotic stewardship efforts to shorten therapy are hampered by the lack of research regarding minimally acceptable durations of treatment for BSIs. Our systematic review and meta-analysis indicate that both inpatients and outpatients with non-*S. aureus *bacteremia secondary to mild to moderate intra-abdominal infection, CAP, or pyelonephritis may be successfully treated with shorter (5 to 7 days) courses of therapy. However, this finding must be interpreted with caution, as only very small numbers of patients and subgroup analyses are currently available for interpretation. A large dedicated trial of treatment duration for bacteremia in severely ill patients is urgently needed to determine the optimal duration of therapy.

## Key messages

• The optimal duration of treatment for bloodstream infections is understudied.

• Available data from bacteremic subgroups of prior randomized controlled trials suggest that shorter-duration therapy (not more than 7 days) may be as effective as longer-duration therapy in achieving clinical cure, microbiologic cure, and survival among most patients with bloodstream infections.

• A large dedicated randomized trial of treatment duration for bacteremia is urgently needed.

## Abbreviations

BSI: bloodstream infection; CAP: community-acquired pneumonia; CI: confidence interval; CRBSI: catheter-related bloodstream infection; ICU: intensive care unit; IDSA: Infectious Diseases Society of America; RCT: randomized controlled trial; SBP: spontaneous bacterial peritonitis; SSTI: skin and soft tissue infection; VAP: ventilator-associated pneumonia.

## Competing interests

The authors declare that they have no competing interests.

## Authors' contributions

All authors contributed to the inception of the research question and study design. TCH executed the main database searches and helped to extract data from individual studies by using prespecified methods determined by all study authors. ND independently reviewed a subset of 1,200 citations and helped to extract data from individual studies by using prespecified methods determined by all study authors. All authors contributed to drafting the manuscript and read and approved the final manuscript.

## Supplementary Material

Additional file 1 **Forest plot for outcome of clinical cure among overall study populations (irrespective of presence or absence of bacteremia) in trials of bacteremia and each of the most common infectious syndromes causing bacteremia (SSTI = skin and soft tissue infection)**. Click here for file

Additional file 2 **Forest plot for outcome of microbiologic cure among overall study populations (irrespective of presence or absence of bacteremia) in trials of bacteremia and each of the most common infectious syndromes causing bacteremia**. Click here for file

Additional file 3 **Forest plot for outcome of survival among overall study populations (irrespective of presence or absence of bacteremia) in trials of bacteremia and each of the most common infectious syndromes causing bacteremia**. Click here for file
