# Supplementary figures and images for: Duration of antibiotic therapy for bacteremia: a systematic review and meta-analysis
Source: Crit Care. 2011 Nov 15;15(6):R267. doi: 10.1186/cc10545 (PMC3388653; doi:10.1186/cc10545)

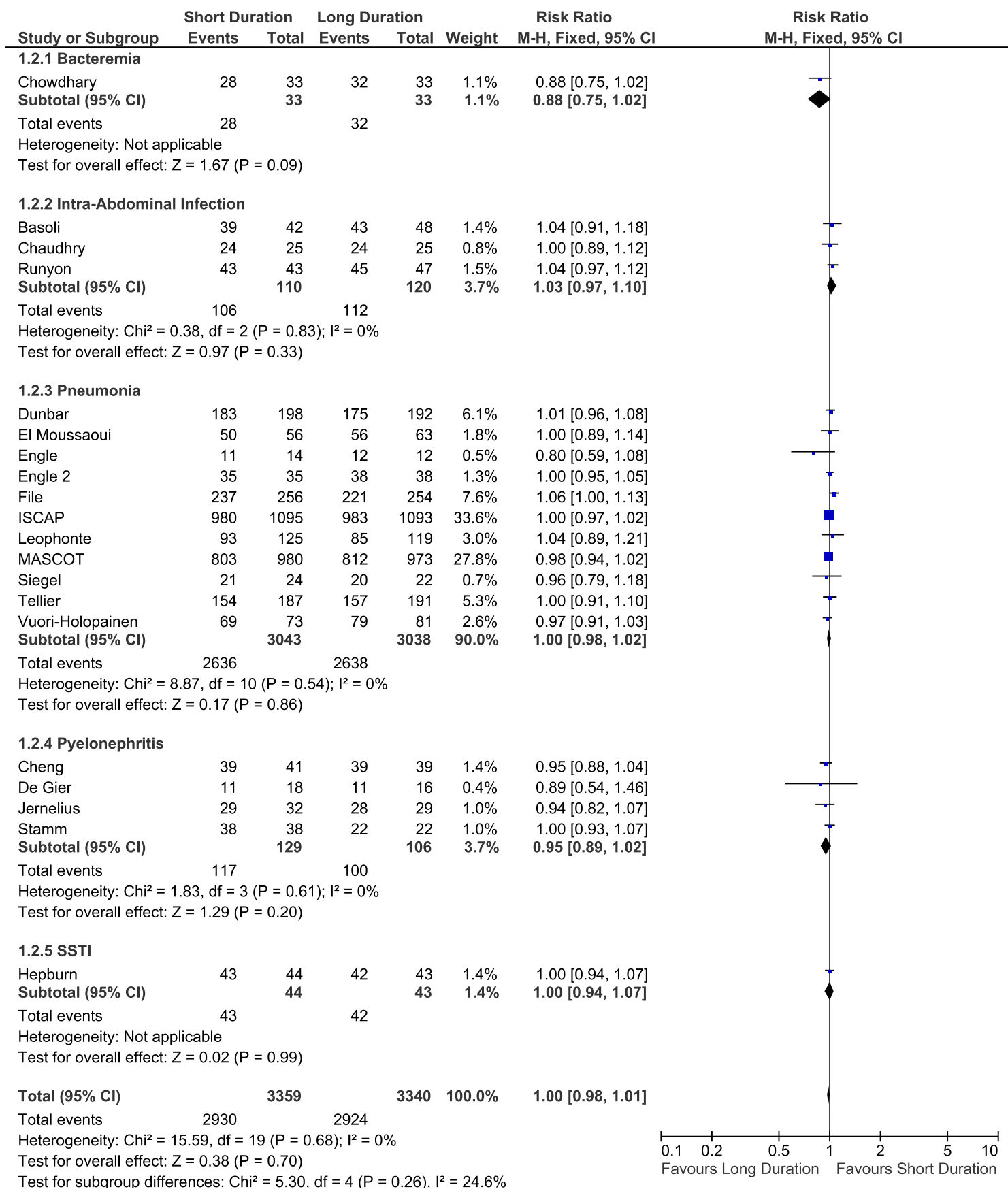

Supplement: Additional file 1 — Forest plot for outcome of clinical cure among overall study populations (irrespective of presence or absence of bacteremia) in trials of bacteremia and each of the most common infectious syndromes causing bacteremia (SSTI = skin and soft tissue infection). [file cc10545-S1.PDF]

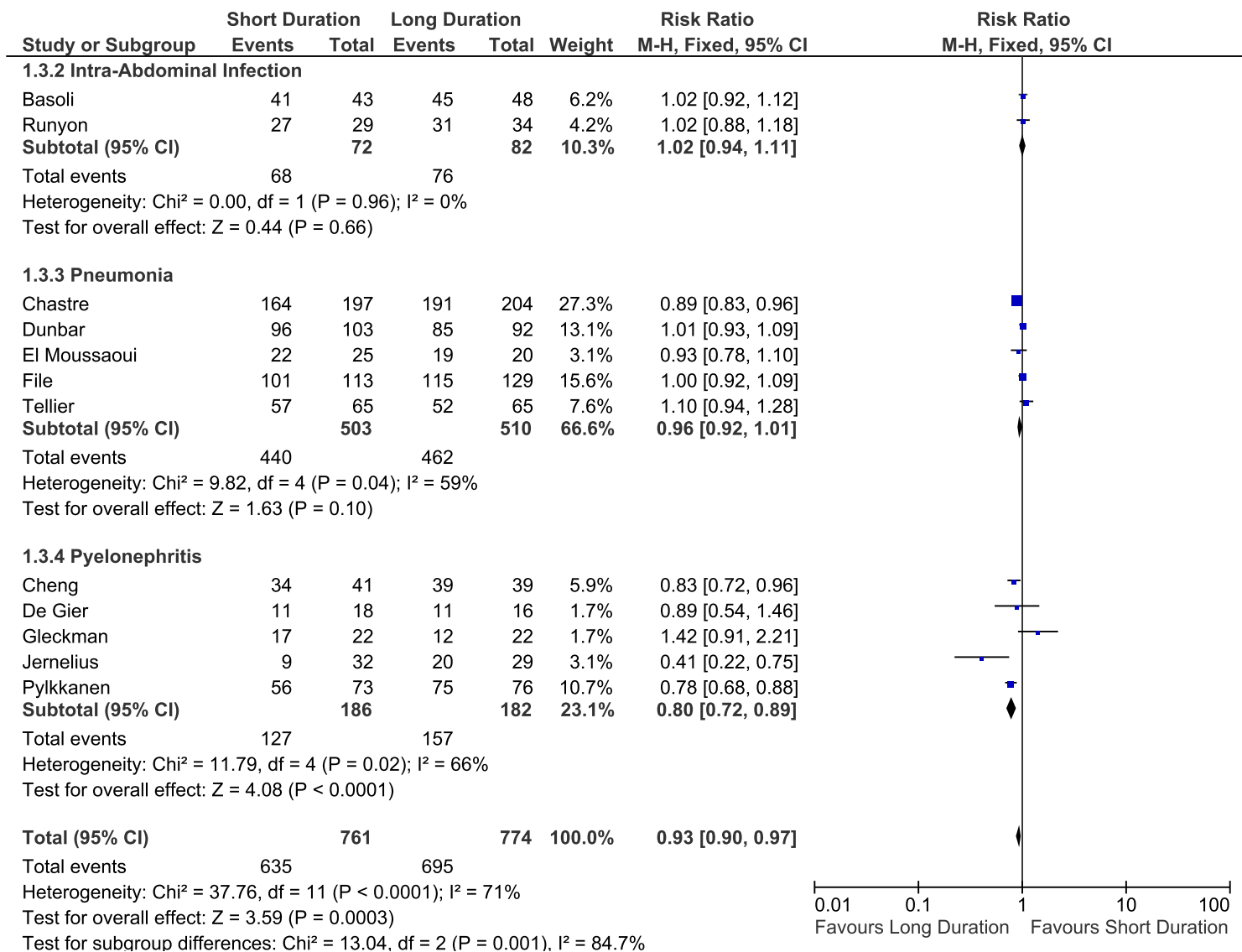

Supplement: Additional file 2 — Forest plot for outcome of microbiologic cure among overall study populations (irrespective of presence or absence of bacteremia) in trials of bacteremia and each of the most common infectious syndromes causing bacteremia. [file cc10545-S2.PDF]

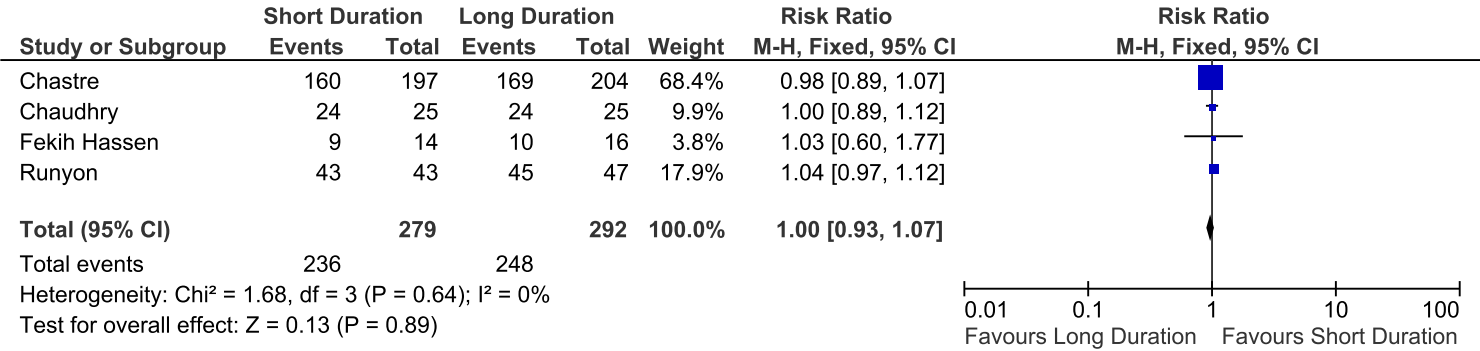

Supplement: Additional file 3 — Forest plot for outcome of survival among overall study populations (irrespective of presence or absence of bacteremia) in trials of bacteremia and each of the most common infectious syndromes causing bacteremia. [file cc10545-S3.PDF]
